# Temperature dependence of the SARS-CoV-2 affinity to human ACE2 determines COVID-19 progression and clinical outcome

**DOI:** 10.1016/j.csbj.2020.12.005

**Published:** 2020-12-16

**Authors:** Zhonghua Zhou, Ziyi Yang, Junxian Ou, Hong Zhang, Qiwei Zhang, Ming Dong, Gong Zhang

**Affiliations:** a MOE Key Laboratory of Tumor Molecular Biology and Key Laboratory of Functional Protein Research of Guangdong Higher Education Institutes, Institute of Life and Health Engineering, College of Life Science and Technology, Jinan University, Guangzhou 510632, China; b Bioland Laboratory, Guangzhou Regenerative Medicine and Health Guangdong Laboratory, Guangzhou, China; c Chi-Biotech Co. Ltd., Shenzhen 518023, China; d Guangdong Provincial Key Laboratory of Tropical Disease Research, School of Public Health, Southern Medical University, Guangzhou, Guangdong 510515, China; e Guangdong Provincial Key Laboratory of Virology, Institute of Medical Microbiology, Jinan University, Guangzhou, Guangdong 510632, China

**Keywords:** SARS-CoV-2, COVID-19, Spike protein, Affinity, Temperature dependence, Structural basis

## Abstract

The SARS-CoV-2 virus and its homolog SARS-CoV penetrate human cells by binding of viral spike protein and human angiotensin converting enzyme II (ACE2). SARS-CoV causes high fever in almost all patients, while SARS-CoV-2 does not. Moreover, analysis of the clinical data revealed that the higher body temperature is a protective factor in COVID-19 patients, making us to hypothesize a temperature-dependent binding affinity of SARS-CoV-2 to human ACE2 receptor. In this study, our molecular dynamics simulation and protein surface plasmon resonance cohesively proved the SARS-CoV-2-ACE2 binding was less affinitive and stable under 40 °C (~18 nM) than the optimum temperature 37 °C (6.2 nM), while SARS-CoV-ACE2 binding was not (6.4 nM vs. 8.5 nM), which evidenced the temperature-dependent affinity and explained that higher temperature is related to better clinical outcome. The decreased infection at higher temperature was also validated by pseudovirus entry assay using Vero and Caco-2 cells. We also demonstrated the structural basis of the distinct temperature-dependence of the two coronaviruses. Furthermore, the meta-analysis revealed a milder inflammatory response happened in the early stage of COVID-19, which explained the low fever tendency of COVID-19 and indicated the co-evolution of the viral protein structure and the inflammatory response. The temperature dependence of the binding affinity also indicated that higher body temperature at early stages might be beneficial to the COVID-19 patients.

## Highlights

• The SARS-CoV-2 virus binds to human ACE2 much weaker at 40 °C than 37 °C.
• The infection efficiency of SARS-CoV-2 is much weaker at high febrile temperature.
• The temperature dependence of viral infection co-evolves with inflammatory response.

## Graphical abstract

**Figure:**
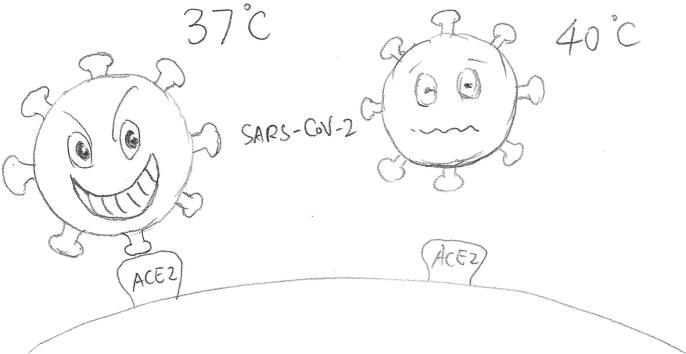

## 1 Introduction

Coronaviruses are the largest group of viruses which belongs to the family Coronaviridae, usually causing respiratory diseases. There are 7 types of coronaviruses in humans, four of which are human coronaviruses (HCoV-NL63, HCoV-229E, HCoV-OC43 and HKU1), which cause limited mild respiratory symptoms, while the other three are highly pathogenic coronaviruses from animals - Severe Acute Respiratory Syndrome Coronavirus (SARS-CoV), Middle East Respiratory Syndrome Coronavirus (MERS-CoV) and 2019 Coronavirus (SARS-CoV-2), which cause severe respiratory disease. SARS-CoV-2 has caused the pandemic Coronavirus Disease 2019 (COVID-19).

For severe COVID-19 patients, the respiratory failure from acute respiratory distress syndrome (ARDS) is the leading cause of mortality [1]. The fatality rate of severe patients can be as high as 67% [2]. Therefore, analyzing the potential factors which may lead to severity is essential in clinical practice. A clinical statistic of 1099 COVID-19 patients showed an interesting conclusion. Among all respiratory symptoms taken into consideration, including body temperature at admission, fever during hospital admission, conjunctival congestion, nasal congestion, headache, cough, sore throat, sputum production, fatigue, hemoptysis, nausea on vomiting, etc., body temperature at admission is the sole statistically significant factor which is not directly relevant to death but prognoses death: the death cases had significant lower temperature (36.8 °C) than the survived cases (37.3 °C) [3], [4]. Another study on the COVID-19 patients in the US also demonstrated that the patients with lower body temperature (36 °C or lower) had significantly higher mortality compared to the patients with higher body temperatures [5].

The genome size of SARS-CoV and SARS-CoV-2 is usually 29 kb. The SARS-CoV-2 shared less than 80% genomic homology to the SARS-CoV, which caused the outbreak of severe acute respiratory syndrome (SARS) in 2002–2003. Coronavirus particles contain four main structural proteins. These are the spike (S), membrane (M), envelope (E), and nucleocapsid (N) proteins, all of which are encoded within the 3′ end of the viral genome. The S protein (~150 kDa) is glycosylated and homotrimers. The trimeric S glycoprotein mediates attachment to the host receptor. Coronaviruses S protein is cleaved into two polypeptides: S1 and S2 [6]. S1 makes up the receptor binding domain of the S protein, while S2 forms the stalk of the spike molecule [7].

Indeed, the invasion of both CoVs into human cells is mediated by the binding of the spike protein (S-protein) RBD domain to the human angiotensin converting enzyme II (ACE2) [8]. However, the clinical symptoms of these two CoVs are largely different. The most perceivable difference is the body temperature of the patients. SARS leads to high fever (>38 °C) in 97% of all cases, making it as a very effective screening marker [9]. In sharp contrast, only 43.1% of the SARS-CoV-2 patients showed fever (≥37.5 °C) when admitted to hospital, among which only 21.7% had a high fever over 38 °C [10]. Recently, many asymptomatic infection cases have been identified, estimated at 17.9%~41.6% of the population [11], [12]. The largely “near-normal” body temperature of the COVID-19 patients sets a great challenge in quick screening of potential patients among crowd. It is estimated that 86% of the COVID-19 infections were undocumented, and thus facilitates the rapid spread of the disease [13].

Temperature may influence the affinity of protein–protein interactions. Therefore, we postulate that the SARS-CoV-2 RBD-ACE2 affinity decreases at high temperatures (>38 °C), but not for the SARS-CoV RBD-ACE2 affinity. If this was true, the viral multiplicity would be delayed if the patient had a high fever, and this delays the progression of viral damage. In this study, we validated this hypothesis via computational and experimental approaches, and provided potential insights for clinical practice.

## 2 Materials and methods

### 2.1 Molecular dynamics simulation

The complex structure of the SARS-CoV-2 S-protein RBD domain and human ACE2 was obtained from Nation Microbiology Data Center (ID: NMDCS0000001) (PDB ID: 6LZG). The complex structure of SARS S-protein RBD domain and human ACE2 was obtained from the PDB database (PDB ID: 2AJF). Molecular dynamics simulation was performed using GROMACS 2019 with the following options and parameters: explicit solvent model, system temperature 37 °C and 40 °C, OPLS/AA all-atoms force field, LINCS restraints. With 2 fs steps, each simulation was performed 100 ns, and each model was simulated three times to generate three independent trajectory replications. Binding free energy (ΔG) was calculated using MM-PBSA method (software downloaded from GitHub: https://github.com/Jerkwin/gmxtool) with the trajectories after structural equilibrium assessed using RMSD (Root Mean Square Deviation)^7^. The B-factors of the C-α of each amino acid, which represent the fluctuation, were derived from the trajectory at equilibrium state. The B-factors were defined as $=\frac{8}{3}\pi^{2}\langle {\left|\Delta r\right|}^{2}\rangle$, where $\langle {\left|\Delta r\right|}^{2}\rangle$ is the mean square atomic displacement relative to the average position [14]. Average structures with b-factor were generated using GROMACS 2019. Structures were visualized using the Pymol software.

### 2.2 Surface plasmon resonance (SPR) experiments

The SPR experiments were performed in a BIAcore T200 instrument (GE, USA). The SARS and SARS-CoV-2 S-proteins were immobilized in the Sensor Chip NTA (GE, USA), respectively, according to the manual protocol. Human ACE2 protein was injected in each experiment in 8 concentrations (3.125, 6.25, 12.5, 25, 50, 100, 200, 400 nM). For each cycle, the absorption phase lasts for 120 s and the dissociation phase lasts for 600 s. After each cycle, the chip was regenerated using 350 mM EDTA and 50 mM NaOH for 120 s, respectively. Blank controls with 0 nM ACE2 were performed, and the blank signals were subtracted from the cycle signals. For each protein, the experiments were performed at 36, 37, 38 and 40 °C, respectively. K_D_ values were calculated via fitting the curves using the software provided with the instrument.

### 2.3 Production and titration of SARS-CoV-2 pseudoviruses

The full-length S gene of SARS-CoV-2 strain Wuhan-Hu-1 (NC_045512.2) was cloned into SARS-CoV-2 Spike vector (PackGene, Guangzhou, China) and confirmed by sequencing. Plasmid of pNL4-3-Luc-R-E and SARS-CoV Spike vector puc-SARS-CoV-spike [15] were donated by Prof. Lu Lu (Fudan University).

Generation of SARS-CoV-2 and SARS-CoV spike HIV-1 backbone pseudovirus was done as previously described with some modifications [16], [17]. Briefly, for SARS--CoV-2 Spike pseudoviruses, 293 T cells were co-transfected with 9 μg pLv-CMV, 6ug psPAX-lentiviral and 6ug pCB-spike, respectively, into 70–90% confluence 150 mm plate. For SARS--CoV Spike pseudoviruses, 293 T cells were co-transfected with 15 μg pNL4-3-Luc-R-E and 15 μg puc-SARS-CoV-spike into 70–90% confluence 150 mm plate. Pseudovirus were harvested 48 h post-transfection, using a 2.5 ml sterile syringe, and subsequently filtered into Falcon or microcentrifuge tubes via a syringe driven 0.45 µm filter. Virus transduced titration was measured by (RT)-qPCR target on WPRE or envelope gene of pseudoviruses through absolute quantification. The titers of the SARS-CoV-2 pseudoviruses were calculated by determining the number of viral transduced DNA per milliliter of viral stock solution using real-time (RT)-qPCR with primers that target WPRE gene: Sense primer: 5′- CACCACCTGTCAGCTCCTTT-3′, anti-sense primer: 5′- ACGGAATTGTCAGTGCCCAA-3′The titers of the SARS-CoV-2 pseudoviruses were calculated by determining the number of viral transduced genomes per milliliter of viral stock solution using real-time (RT)-qPCR with primers that target envelope gene: Sense primer: 5′-TAGTAGGAGGCTTGGTAGG-3′, anti-sense primer: 5′-AGGTGGGTCTGAAACGATA-3′.

### 2.4 SARS-CoV-2 and SARS-CoV spike-mediated pseudovirus entry assay

To test temperature-dependent viral entry, Vero E6 and Caco2 cells (5 × 10^4^) grown in 24-well plates were respectively infected with equal 2.1 × 10^3^ TU pseudovirus in 250 μL DMEM. The invasion was performed under 37 °C and 40 °C, respectively. The virus in the medium was then washed away, and the cells were then cultured at 37 °C. The cells were collected 3 h post-infection or added 150ul fresh DMEM medium 6 h post-infection. Relative fold change of infected virus calculated by the RNA and DNA copies number of the target envelope gene have been measured 3 h and 24 h post-infection. RNA was extracted 3 h post-infection using Trizol and reverse transcribed with Reverse Transcriptase M-MLV (RNase H free) (Takara, Japan). The relevant fold of the entry pseudoviruses RNA and transduced DNA copies were calculated using real-time (RT)-qPCR with TB Green™ Premix Ex Taq™ II (Tli RNaseH Plus) (Takara, Japan).

The relevant fold of the pseudoviruses were calculated by determining the number of viral transduced genomes per using real-time (RT)-qPCR and qPCR with primers and a probe that target envelope gene.

## 3 Results

### 3.1 Clinical hint of the temperature-dependent invasion

**Fig. 1:**
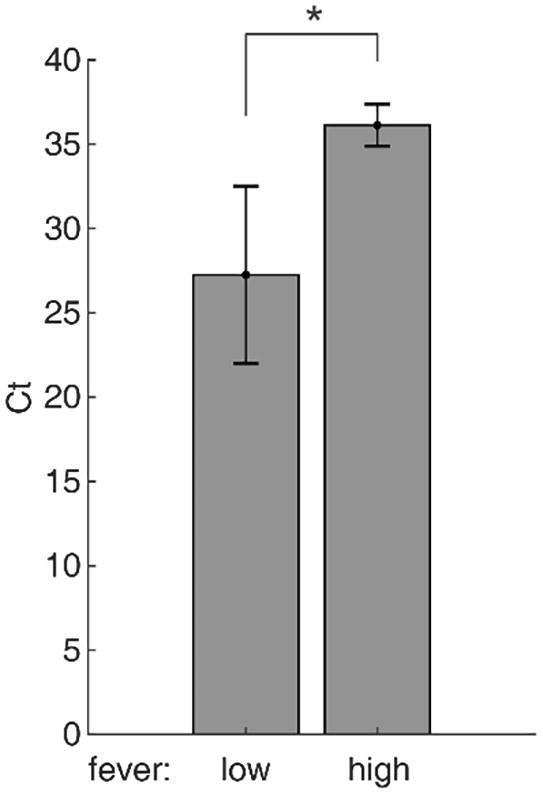
RT-PCR Ct values of the low fever and high fever patients. Data are presented as mean ± SE.

If the high temperature weakens the viral invasion, the patients with high fever would have lower viral load. To test this postulation, we re-analyzed the published data from Zou et al. [18]. Viral load was evaluated by the Ct values (cycles at threshold) in the RT-PCR experiments. Lower Ct values represent higher viral load. The febrile patients were divided into two groups: low fever (37.5 ~ 38 °C) and high fever (>38 °C). We took the throat swab result to maximize the number of patients in each group. The Ct values of the high fever group is significantly higher than the low fever group (*P* = 0.0444, single-sided Mann-Whitney *U* test, Fig. 1), indicating that the viral load in low fever is significantly higher than the high fever group, i.e. the clinical data supports the temperature-dependent invasion hypothesis.

### 3.2 Temperature dependence of RBD-ACE2 binding affinity

**Fig. 2:**
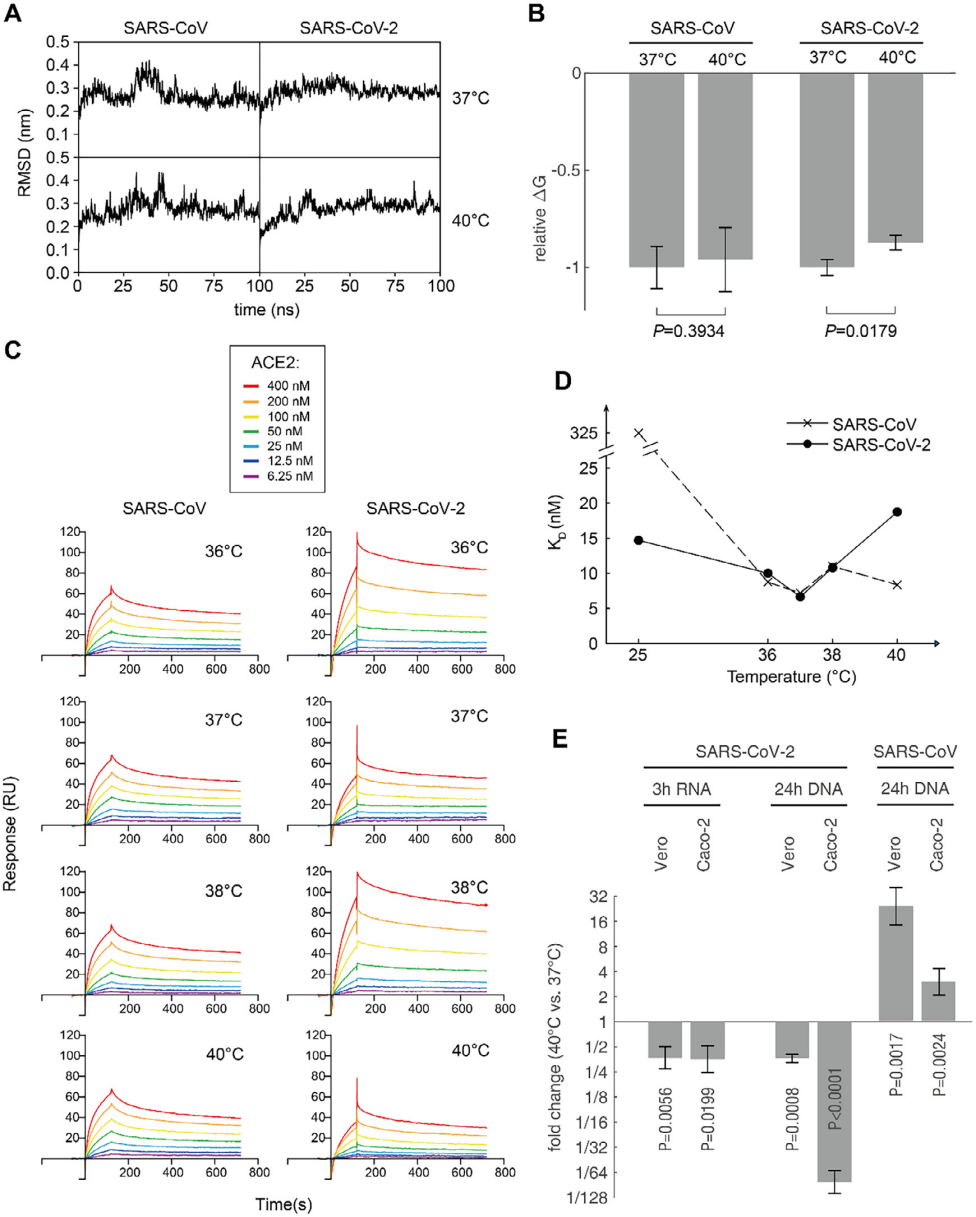
Computational and experimental assessment of RBD-ACE2 binding affinity. (A) The typical RMSD curves of 100 ns molecular dynamics simulation trajectories of the RBD-ACE2 complexes at 37 °C and 40 °C, respectively. (B) The relative binding free energy (ΔG) normalized using the ΔG at 37 °C of each CoV, respectively. Lower ΔG means higher affinity. Data are presented as mean ± SD (three independent replicates). (C) Surface plasmon resonance (SPR) assay of the S-proteins of the two CoVs binding to human ACE2, at different temperatures. Details of the binding data are summarized in Supplementary Table S1. (D) The K_D_ values measured using SPR experiments at different temperatures. The data points at 25 °C are taken from Wrapp et al. [19] (E) S protein-containing pseudovirus infection assay. ACE2-overexpressed Vero and Caco-2 cells were used as hosts. The invasion was performed at 37 °C and 40 °C, and the cells were then washed to remove the unpenetrated virus. The cells were then cultured at 37 °C. The penetrated viral RNA was measured 3 h post infection, and the integrated viral genome into the host was measured by qPCR.

The first step of SARS-CoV-2 invasion was to bind human ACE2 with RBD of the spike protein, and this binding affinity can be affected by temperature. To investigate the temperature dependence of RBD-ACE2 binding affinity, we first performed molecular dynamics simulation for the complex of human ACE2 bound with SARS-CoV-2 RBD and SARS RBD, respectively. The simulation was performed at the temperature settings 37 °C and 40 °C, respectively. Each scenario was simulated for 100 ns and triplicated. The RMSD curves of the complex reached equilibrium after 50 ns (Fig. 2A). The binding energy (ΔG) of SARS-CoV-2 at 40 °C was significantly higher than that at 37 °C (*P* = 0.0179, Student *t*-test), while SARS showed no significant difference at both temperatures (Fig. 2B). This indicated that the human ACE2 binds to SARS-CoV-2 weaker at high temperatures, while its affinity to SARS remains unchanged.

To experimentally validate the *in silico* results, we performed surface plasmon resonance (SPR) experiment to measure the binding affinity of the full-length S-proteins and human ACE2 from 36 °C to 40 °C (Fig. 2C). From 36 °C to 38 °C, the S-proteins of the two coronaviruses bind to human ACE2 with similar affinity. However, at 40 °C, the affinity of SARS-CoV-2 S-protein significantly decreased, as the equilibrium dissociation constant (K_D_) significantly increased for almost 3 times (*P* = 0.0002, Student *t*-test), while the SARS RBD maintained the similar K_D_ as at lower temperatures (Fig. 2D). These data experimentally validated the computational results and our hypothesis.

To validate the *in vitro* results, we performed *in vivo* experiment to investigate the invasion efficiency of the SARS-CoV-2 under different temperatures. ACE2-overexpressed Vero and Caco-2 cells were subjected to S protein-containing pseudovirus infection assay, respectively. The infection efficiency can be assessed in two dimensions: a) the viral RNA inside the host cells shortly after the invasion, which is the direct measure of penetrated virus; b) the copy number of integrated lentivirus genome into the host genome, which represents the number of functional virus penetrated the cell. Three hours post infection, qRT-PCR revealed that the penetrated viral RNA at 40 °C reached only 37.0% and 35.5% in Vero and Caco-2 cells compared to which at 37 °C, respectively. 24 h post infection, the integrated viral genome at 40 °C reached only 36.5% and 1.20% in Vero and Caco-2 cells compared to which at 37 °C, respectively. (Fig. 2E) The decrease of all experiments at 40 °C were statistically significant (*P* < 0.05, *t*-test), demonstrating that the lower affinity of SARS-CoV-2 RBD to ACE2 at high temperature delays the viral invasion. In contrast, SARS-CoV showed higher copy number after infection under 40 °C than 37 °C (Fig. 2E), indicating that higher temperature facilitates the infection of SARS-CoV.

### 3.3 Structural basis of the temperature-dependent affinity of SARS-CoV-2

**Fig. 3:**
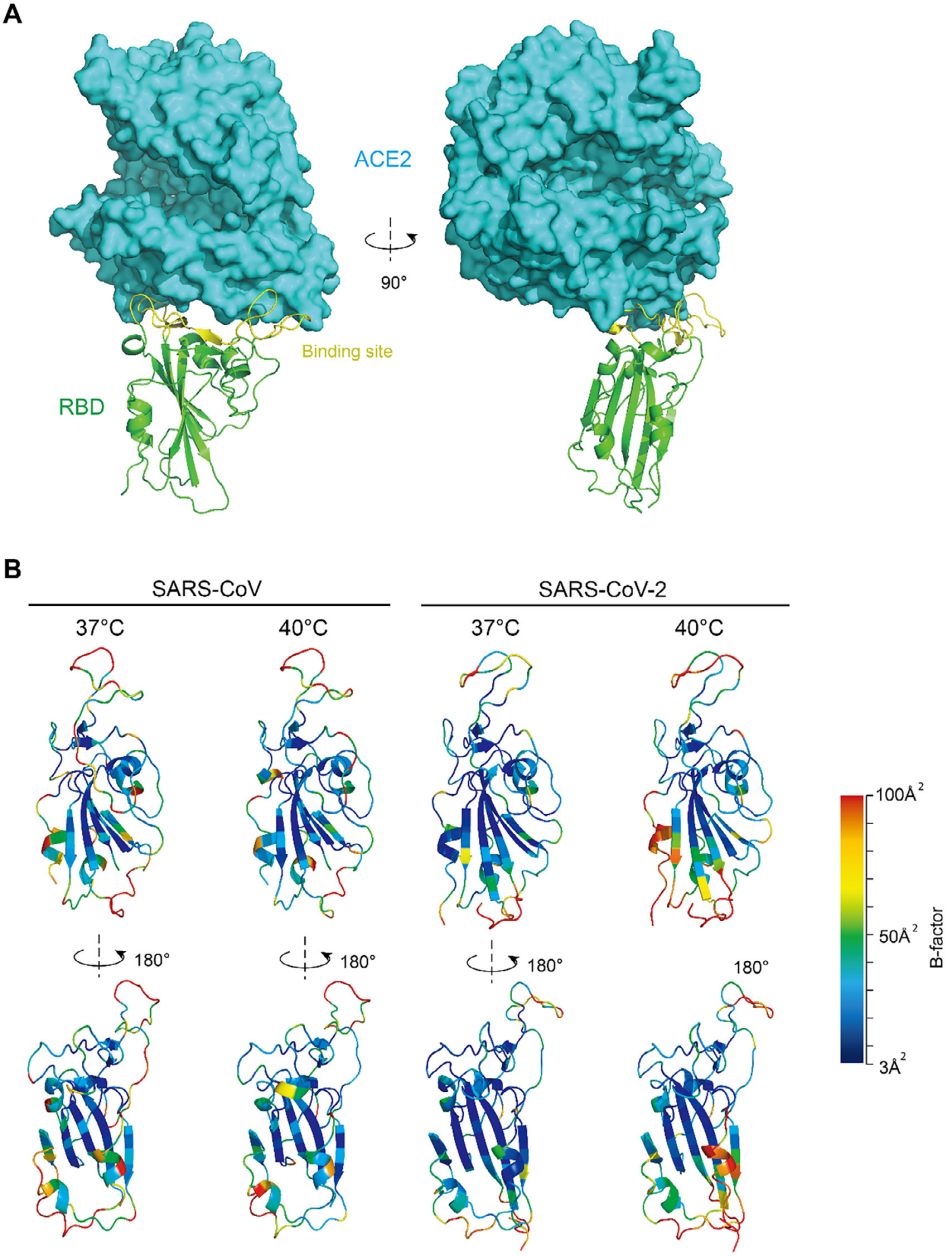
Structural basis of the temperature-dependence of the binding affinity. (A) Structure of SARS-CoV-2 RBD and human ACE2 complex. (B) The RBD backbone colored according to the B-factors of each amino acid. Higher B-factor means larger fluctuation, i.e. more flexible.

RBD is the domain which directly interacts with human ACE2. It is relatively independent of the S protein in structure. The center of this domain is a scaffold which is built by beta-sheet structure. This scaffold stabilizes the entire domain, especially the binding site, which is mainly in random-coil conformation (Fig. 3A). The flexibility at equilibrium reflects the rigidity of the structure, which can be assessed by the B-factor of the C-alpha atoms of each amino acid [14]. More rigid structure consolidates the binding affinity. When elevating temperature from 37 °C to 40 °C, the fluctuation of the SARS-CoV-2 RBD at binding state remarkably increases, especially at and near the binding site and the lower part of the scaffold. In sharp contrast, the SARS RBD showed almost no difference (Fig. 3B). These results explained the pattern of the temperature dependence of the two RBDs.

## 4 Discussion

In this study, we demonstrated the temperature dependence of binding affinity of SARS-CoV-2 S-protein to human ACE2. The binding is optimized at 37 °C, and is significantly decreased at 40 °C because of enhance fluctuation in the RBD domain. This characteristic is distinguished from the homologous SARS-CoV, which remain similar binding affinity at high body temperature. This finding may provide key insights in both clinical and biochemical aspects.

High fever is a vigorous response against the viral infection. To avoid organ failure caused by continuous high fever (>38 ~ 38.5 °C), febrifuge treatments are often deployed immediately when a high fever is detected to decrease the body temperature. However, high temperature at early stage of SARS-CoV-2 infection impairs the binding to human cells (Fig. 1) and thus retard the viral progression, leading to a lower viral load in patients (Fig. 3). This coincide with a previous model that the K_D_ of virus and host cells negatively correlate to the viral multiplicity [20]. Lower viral load at early stage will delay the lesion of multiple organs and thus make time for the immune system to kill and clear the virus before severe failure of multiple organs. This explains the clinical outcomes: the higher body temperature at admission is the sole factor among the respiratory symptoms which significantly prognoses less fatality [10]; about 80% of the young children patients had high fever, and their pneumonia were not so severe as adult patients, among which only 21% had high fever [21], [22], [23], [24], [25].

We investigated differences in COVID-19 and SARS patients’ innate immune response to explain the tendency of low fever in COVID-19 by analyzing the clinical data of 2300 patients. Fever, a manifestation of physical inflammatory response, is caused by abnormal production and release of cytokines and chemokine after virus invasion, especially when immune cells such as macrophages, dendritic cells and lymphocytes were infected [26]. The SARS-CoV-2 causes milder inflammatory response (cytokines release) than SARS-CoV, which partly explains the low degree fever in the early stage. SARS-CoV induced pro-inflammatory cytokines production and pyroptosis in macrophages and lymphocytes [27], but whether the SARS-CoV-2 has the same function and mechanism is still unknown. The distinct immune indicators of the two CoVs indicated fundamentally different immune response pathways of these two diseases, which is worth for further exploration. Therefore, referring the knowledge learned from the immune response of SARS and MERS should be very careful in COVID-19 studies and treatment.

The milder immune response of the COVID-19 patients at early stages optimizes the viral progression in the patients. It also coincides to the fact that most death cases did not show severe symptoms at early stages; however, their conditions deteriorated suddenly in the later stages of the disease or in the process of recovery. At late stages, the cytokine storm was thought to be the cause of the ARDS [28]. Although the mechanism of massive release of cytokines at late stages is still not clear for COVID-19 [28], the higher number of neutrophils, lymphocytes and monocytes than SARS patients were stimulated by the excessive cytokines and thus causes sudden and severe lesions, leading to ARDS. This coincides to the opinion that the secondary haemophagocytic lymphohistiocytosis (sHLH), a hyperinflammatory symdrome, triggers the ARDS [29]. Excessive cytokines will also elevate the body temperature at late stage. This explains the fact that the highest body temperature during hospital admission is not related to the clinical outcome [10]. Taken together, these knowledges emphasize the importance of controlling the viral infection and progression at early stage before the hyperinflammatory sHLH. Making use of the temperature-dependence of affinity might be a simple and effective strategy: the febrifuge should probably not be generally applied at early stage of SARS-CoV-2 stage; treatments to temporarily elevate body temperature might be also considered.

Both SARS and COVID-19 are self-limited diseases, which means that the pathogen need to continuously infect healthy individuals for survival. Coincidently, both SARS-CoV and SARS-CoV-2 have the lowest K_D_ (i.e. the highest affinity) at 37 °C (Fig. 1D), which is the normal human body temperature. This indicates that they are optimized to infect healthy people, which might be a feature created by evolution. However, after a successful infection, these two viruses stimulate inflammatory response differently. The SARS-CoV-2 tend to keep the cytokines at lower level to keep the body temperature relatively; otherwise, its progression would be retarded by high fever. However, the binding affinity of SARS-CoV is optimized at high temperature; therefore, the lower temperature would retard its progression. The specific structural nature of the S-protein RBD domain determines the temperature-dependent structural rigidity and thus the temperature-dependent affinity. This drives the different direction of evolution of these two viruses. In another aspect, besides the primates, most other mammals and birds demonstrate normal body temperature higher or lower than 37 °C [30]. To a certain extent, this implicates that both SARS-CoV and SARS-CoV-2 viruses have been adequately adapted to the human host before the outbreak. This echoes those opinions that the SARS-CoV-2 might have been spread among human society long before the outbreak in Wuhan, China [31].

## Funding statement

This work was supported by grants from the National Key Research and Development Program of China (2017YFA0505001/2018YFC0910200/2018YFE0204503), the Guangdong Key Research and Development Program (2019B020226001), the 10.13039/501100003453Natural Science Foundation of Guangdong Province, China (2018B030312010), Bioland Laboratory (Guangzhou Regenerative Medicine and Health Guangdong Laboratory) (1102101203) and the Guangzhou Healthcare Collaborative Innovation Major Project (201803040004 and 201803040007).

## CRediT authorship contribution statement

**Zhonghua Zhou:** Methodology, Software, Data curation, Formal analysis, Visualization. **Ziyi Yang:** Data curation, Formal analysis, Writing - original draft. **Junxian Ou:** Methodology, Validation. **Hong Zhang:** Methodology, Data curation, Visualization. **Qiwei Zhang:** Conceptualization, Project administration, Fuding acquisition, Supervision, Resource, Writing - review & editing. **Ming Dong:** Conceptualization, Project administration, Funding acquisition, Writing - original draft. **Gong Zhang:** Conceptualization, Project administration, Funding acquisition, Supervision, Visualization, Data curation, Writing - original draft, Writing - review & editing.

## Declaration of Competing Interest

The authors declare that they have no known competing financial interests or personal relationships that could have appeared to influence the work reported in this paper.
