# Supplementary material for: Temperature dependence of the SARS-CoV-2 affinity to human ACE2 determines COVID-19 progression and clinical outcome
Source: Comput Struct Biotechnol J. 2020 Dec 16;19:161–7. doi: 10.1016/j.csbj.2020.12.005 (PMC7738279; doi:10.1016/j.csbj.2020.12.005)
Supplement: Supplementary data 1 [file mmc1.docx]

**Temperature Dependence of the SARS-CoV-2 affinity to human ACE2 determines COVID-19 progression and clinical outcome**

**SUPPLEMENTARY MATERIALS**

**Table S1**: Binding parameters calculated from the SPR experiments. k_a_ = association constant; k_d_ = dissociation constant; K_D_ = equilibrium dissociation constant.

| Temperature (°C) | S-protein | k_a_ (1/Ms) | SE (k_a_) | k_d_ (1/s) | SE (k_d_) | K_D_ (M) |
| --- | --- | --- | --- | --- | --- | --- |
| 36 | SARS | 7.39E+04 | 1.70E+02 | 6.47E-04 | 3.10E-06 | 8.75E-09 |
| 36 | COVID-19 | 4.08E+04 | 6.10E+01 | 4.13E-04 | 1.60E-06 | 1.01E-08 |
| 37 | SARS | 8.80E+04 | 1.80E+02 | 6.32E-04 | 2.90E-06 | 7.18E-09 |
| 37 | COVID-19 | 6.92E+04 | 1.30E+02 | 4.61E-04 | 2.60E-06 | 6.66E-09 |
| 38 | SARS | 6.03E+04 | 1.30E+02 | 6.62E-04 | 2.80E-06 | 1.10E-08 |
| 38 | COVID-19 | 4.24E+04 | 5.70E+01 | 4.59E-04 | 1.50E-06 | 1.08E-08 |
| 40 | SARS | 8.65E+04 | 1.70E+02 | 7.23E-04 | 2.80E-06 | 8.36E-09 |
| 40 | COVID-19 | 4.53E+04 | 1.30E+02 | 8.49E-04 | 3.20E-06 | 1.87E-08 |
